# High Incidence of Distant Metastasis Is Associated With Histopathological Subtype of Pediatric Papillary Thyroid Cancer - a Retrospective Analysis Based on SEER

**DOI:** 10.3389/fendo.2021.760901

**Published:** 2021-11-11

**Authors:** Xue Zeng, Zhihong Wang, Zhiqiang Gui, Jingzhe Xiang, Mengsu Cao, Wei Sun, Liang He, Wenwu Dong, Jiapeng Huang, Dalin Zhang, Chengzhou Lv, Ting Zhang, Liang Shao, Ping Zhang, Hao Zhang

**Affiliations:** Department of Thyroid Surgery, The First Hospital of China Medical University, Shenyang, China

**Keywords:** papillary thyroid carcinoma (PCT), histopathological subtype, distant metastasis, pediatrics - children, SEER, FVPTC, CPTC

## Abstract

**Objective:**

Children with papillary thyroid cancer (PTC) have a higher invasive rate and distant metastasis rate, but the mortality rate is lower with unknown reasons. The majority of PTC cases comprise classical papillary thyroid carcinoma (CPTC) and follicular variant papillary thyroid carcinoma (FVPTC). This study aimed to determine the relationship between histopathological subtype and rate of distant metastasis and investigate factors influencing distant metastasis in pediatric PTC.

**Methods:**

A total of 102,981 PTC patients were recruited from SEER registry, 2004-2015. Proportion of distant metastasis between children (≤18 years) and adults with different histopathological subtypes was compared by propensity score matching. The cut-off age for distant metastasis in children was calculated by receiver operating characteristic (ROC) curve, and the risk factors for distant metastasis in pediatric patients were analyzed by logistic regression models.

**Results:**

Among the 1,484 children and 101,497 adults included in the study, the incidence of CPTC patients with distant metastasis in children was higher than that in adults (*p*<0.001). The ROC curve was calculated, which yielded a cut-off age for distant metastasis in CPTC children as 16 years old. In CPTC, the proportion of young children (2-16 years) with distant metastasis was higher than that of adolescents (17-18 years) and adults (>18 years) (both *p*<0.001). While there was no such trend in FVPTC. In young children (2-16 years), the incidence of CPTC with distant metastasis was higher than FVPTC (*p*=0.006). There was no difference between the proportion of CPTC and FVPTC with distant metastasis in adolescents (17-18 years) and adults. Logistic regression models revealed that extrathyroidal extension, lymph node metastasis and CPTC histopathological subtype were risk factors for distant metastasis in young children aged 2 -16 years.

**Conclusions:**

In CPTC, the incidence of distant metastasis in young children (2-16 years) was significantly higher than that in adolescents (17-18 years) and adults (>18 years). In patients with distant metastasis aged 2-16 years, the proportion of CPTC was higer than that of FVPTC. Extrathyroidal extension, lymph node metastasis, and CPTC histopathological subtype were risk factors for distant metastasis in young children aged 2-16 years.

## Introduction

Thyroid cancer is the most common endocrine cancer in the pediatric population (1). In a cross-sectional study based on the Surveillance, Epidemiology, and End Results (SEER) database including individuals younger than 20 years who had a diagnosis of thyroid cancer, the incidence of pediatric thyroid cancer increased by 1.1% per year from 1973 to 2006, with a significant increase of 9.5% per year from 2006 to 2013 (2). Compared to adult thyroid carcinoma, the prognosis of pediatric papillary thyroid cancer is generally fair (3). The reported mortality rate of pediatric papillary thyroid cancer (PTC) is very low with a higher rate of distant metastasis (DM) in most series despite more advanced disease at presentation and a higher risk of recurrence (4–6). A study involving patients aged 12 to 83 years showed that approximately half of patients with well-differentiated thyroid cancer with DM die of disease within 5 years of initial diagnosis despite thyroid surgery and radioactive iodine (RAI) (7). Indeed, the presence of DM is reported to be an independent predictor for poor overall survival (8). Therefore in a disease with a low mortality rate such as PTC, it is critical to identify tumors at initial presentation that are at risk of developing DM to assist in clinical decision making. Despite incidence of DM of PTC in children (≤18 years) was higher than in adults (>18 years), the American Thyroid Association Guidelines for the Treatment of Thyroid Nodules and Differentiated Thyroid Carcinoma do not differentiate children from adults in diagnosis and treatment, thereby calling for further studies to guide treatment strategies. Pediatric PTC patients aged less than 18 years might benefit from tailored disease management by cut-off age and expect better prognosis.

Some published studies have suggested that the frequency of DM was different among the various histopathological subtypes (8), the majority of PTC cases comprise classical papillary thyroid carcinoma (CPTC) and follicular variant papillary thyroid carcinoma (FVPTC) histopathological subtypes (9), which account for 67-74% and 26-30% of cases, respectively (9, 10). According to previous reports, extrathyroidal extension (ETE) and cervical lymph node metastases (LNM) are more common in CPTC than in FVPTC (11). The current consensus among thyroid academia is that there are only a few differences between CPTC and FVPTC. Moreover, the overall management of the two malignancies is similar, and patients with CPTC and FVPTC have similar long-term outcome (11). However, despite these similarities, minimal ETE and aggressive histopathological features, such as thyroid capsule infiltration, are significantly more common in CPTC than in FVPTC (11). Besides, patients with mETE showed significantly higher rates of lymph node metastases in the neck (12) and all levels of ETE, including microscopic ETE, were associated with increased risk for lymph node metastasis and DM (13). Thus, we propose the following hypothesis: The high DM rate in pediatric PTC aged less than 18 years old is associated with histopathological subtype.

Although there were some reports focusing on those young children had a greater degree of ETE and lymph node involvement than adolescents and were more prone to DM (4). Due to the lack of pediatric patients who are necessary to determine the explanation for the differences in clinicopathological outcomes observed in pediatric PTC patients, the focus on the reason why young children and adolescent patients show this difference has been rarely explored. More unfortunately, little attention was paid to differences in pathological subtypes among young children and adolescents. To assess our hypothesis and calculate the cut-off age for DM in children, as well as investigate factors influencing distant metastasis in pediatric PTC, we conducted a retrospective analysis at the first time using a population-based data set with a large sample.

## Materials and Methods

### Data Source

Data for this study were extracted from the National Cancer Institute’s SEER data (Surveillance, Epidemiology, and End Results), which is one of the most representative large oncology registry databases in North America, covering 34.6% of the U.S. population and collecting case information from 18 population-based cancer registries (14).

### Study Participants

The study cohort included patients diagnosed with PTC between 2004 and 2015. All included patients were identified using histopathological codes of the International Classification of Disease for Oncology, Third Revision (ICD-O-3). Histopathological codes were listed below CPTC included 8050/3 (papillary carcinoma not otherwise specified, NOS), 8260/3 (papillary adenocarcinoma, NOS), and 8343/3 (papillary carcinoma, encapsulated); FVPTC included 8340/3 (papillary carcinoma, follicular variant); 8342/3 (papillary carcinoma, oxyphilic cell), 8344/3 (papillary carcinoma, columnar cell), and 8450/3 (nonencapsulated sclerosing carcinoma) were classified as other (15). Exclusion criteria for this study included: (1) age or race unknown; (2) incomplete/missing information on tumor size, tumor invasion, multifocal, LNM, or DM. This study was based on the American Joint Committee on Cancer (AJCC) (Eighth Edition). Data in the SEER database at the time of extraction was based on AJCC (Sixth Edition) and AJCC (Seventh Edition). We performed a second extraction of the data, converting variables defined by the AJCC (Sixth Edition and Seventh Edition) into AJCC (Eighth Edition). Our analysis included demographic variables: sex; age at diagnosis; ethnicity; histopathological subtype; tumor size; ETE; LNM; DM; foci; cancer-specific survival (CSS); and overall survival (OS); survival months.

Age was categorized as ≤18 years and >18 years. The patients aged ≤ 18 years were regarded as children and patients aged >18 years were regarded as adults. Ethnicity was categorized according to the record in the SEER database as Black, White and other. Histopathological subtype was based on ICD-O-3. Tumor size less than 15cm are preserved. ETE was based on “CS extension (2004-2015)” codes. DM and LNM were based on AJCC (Eighth Edition). Foci was based on “CS site-specific factor 1” codes.

### Statistical Analysis

The database materials were obtained by SEER*Stat 8.3.9 software, processed by WPS 2.7.1 software and statistically analyzed by SPSS 26.0 (IBM) software. Patient information, including demographic data and cancer-related information, was compared with histopathological subtypes. In our data, only two histopathological subtypes, CPTC and FVPTC, were present in patients ≤ 18 years old with DM. Therefore, this study focused on CPTC and FVPTC. Categorical variables were reported as frequency and proportion. We used the chi-square test or Fisher’s exact test to compare these variables. To minimize selection bias, propensity score matching (PSM) was performed on ETE and LNM when comparing the proportion of DM in children and adults with different histopathological subtypes. The 1:1 matching scheme was used for matching, and a caliper of 0.05 SD for the probit value. The age cut-points at which CPTC and FVPTC developed DM in children were calculated by receiver operating characteristic (ROC) curve. Logistic regression analysis was used to calculate the risk factors for DM in children and regression coefficient (B), odds ratio (OR), the 95% confidence interval (CI) was used for reporting. Bilateral *p* value <0.05 was considered statistically significant difference. Variables with *p* value <0.05 in univariate analysis were included in multivariate analysis.

## Results

### Demographic and Clinicopathological Characteristics

There were 102,981 patients diagnosed with PTC between 2004 and 2015, who met the inclusion criteria. **Table 1** summarizes the demographic and clinicopathological features of these patients. A total of 101,497 adult patients aged >18 years (98.56%) and 1,484 pediatric patients aged ≤18 years (1.44%) were included. 79,431 (77.13%) were female and 23,550 (22.87%) were male. Of these, 67,614 (65.66%) were CPTC patients, 33,862 (32.88%) were FVPTC patients, and 1,505 (1.46%) were patients with other histopathological subtypes. DM occurred in 999 patients (0.97%), ETE occurred in 18,204 patients (17.68%), LNM occurred in 24,636 patients (23.92%), and 43,804 patients (42.54%) had multifocal tumors. CSS was 98.84% and OS was 92.79%.

**Table 1:** Baseline clinicopathological characteristics of patients with PTC.

	All patients [no. (%)]	Children [no. (%)]	Adults [no. (%)]	P-value
No. of patients	102,981 (100.00)	1,484 (1.44)	101,497 (98.56)	
Gender				<0.001
Female	79,431 (77.13)	1,224 (82.48)	78,207 (77.05)	
Male	23550 (22.87)	260 (17.52)	23,290 (22.95)	
Ethnicity				=0.003
White	85,052 (82.59)	1,263 (85.10)	83,789 (82.56)	
Black	6,522 (6.33)	63 (4.25)	6,459 (6.36)	
Other	11,407 (11.08)	158 (10.65)	11,249 (11.08)	
Histological subtype				<0.001
CPTC	67,614 (65.66)	1,083 (72.98)	66,531 (65.55)	
FVPTC	33,862 (32.88)	388 (26.14)	33,474 (32.98)	
Other	1,505 (1.46)	13 (0.88)	1492 (1.47)	
DM				<0.001
Presence	999 (0.97)	41 (2.76)	958 (0.94)	
Absence	101,982 (99.03)	1,443 (97.24)	100,539 (99.06)	
ETE				<0.001
Presence	18,204 (17.68)	380 (25.61)	17,824 (17.56)	
Absence	84,777 (82.32)	1,104 (74.39)	83,673 (82.44)	
LNM				<0.001
Presence	24,636 (23.92)	774 (52.16)	23,862 (23.51)	
Absence	78,345 (76.08)	710 (47.84)	77,635 (76.49)	
Foci				0.15
Solitary	59,177 (57.46)	880 (59.30)	58,297 (57.44)	
Multifocal	43,804 (42.54)	604 (40.70)	43,200 (42.56)	
CSS				=0.001
Alive/Dead (other causes)	101,785 (98.84)	1,481 (99.80)	100,304 (98.82)	
Thyroid cancer	1,196 (1.16)	3 (0.20)	1,193 (1.18)	
OS				<0.001
Alive	95,551 (92.79)	1,472 (99.19)	94,079 (92.69)	
Dead	7,430 (7.21)	12 (0.81)	7,418 (7.31)	
Survival months (mean ± SD)	79.33 ± 41.53	83.52 ± 41.21	79.27 ± 41.53	<0.001

PTC, papillary thyroid carcinoma; CPTC, classical papillary thyroid carcinoma; FVPTC, follicular variant papillary thyroid carcinoma; DM, distant metastasis; ETE, extrathyroidal extension; LNM, lymph node metastasis; CSS, cancer-specific survival; OS, overall survival; SD, standard deviation.

**Table 2** describes the demographics and clinicopathological features of PTC patients with DM. DM occurred in a total of 999 patients, of whom 41 (4.10%) were children (≤18 years) and 958 (95.90%) were adults (>18 years). Of the pediatric patients with DM, 36 (87.80%) were CPTC patients, 5 (12.20%) were FVPTC patients, and no DM was found in pediatric patients with other histopathological subtype. 28 (68.29%) patients had ETE, 40 (97.56%) patients had LNM, and 25 (60.98%) patients had multifocal tumor, while no patient died. Of the adult patients with DM, 617 (64.41%) were CPTC, 302 (31.52%) were FVPTC, 39 (4.07%) were other histopathological subtypes, 612 (63.88%) had ETE, 629 (65.66%) had LNM, 502 (52.40%) had multifocal tumors, and 451 (47.08%) died, of which 302 (31.52%) died of PTC.

**Table 2:** Baseline clinicopathological characteristics of PTC patients with distant metastasis.

	Children [no. (%)]	Adults [no. (%)]	*P*-value
No. of patients	41 (4.10)	958 (95.90)	
Gender			=0.009
Female	30 (73.17)	501 (52.30)	
Male	11 (26.83)	457 (47.70)	
Ethnicity			0.675
White	34 (82.92)	738 (77.04)	
Black	2 (4.88)	67 (6.99)	
Other	5 (12.20)	153 (15.97)	
Histological subtype			=0.008
CPTC	36 (87.80)	617 (64.41)	
FVPTC	5 (12.20)	302 (31.52)	
Other	0 (0.00)	39 (4.07)	
ETE			0.564
Presence	28 (68.29)	612 (63.88)	
Absence	13 (31.71)	346 (36.12)	
LNM			<0.001
Presence	40 (97.56)	629 (65.66)	
Absence	1 (2.44)	329 (34.34)	
Foci			0.281
Solitary	16 (39.02)	456 (47.60)	
Multifocal	25 (60.98)	502 (52.40)	
CSS			<0.001
Alive/Dead (other causes)	41 (100.00)	656 (68.48)	
Thyroid cancer	0 (0.00)	302 (31.52)	
OS			<0.001
Alive	41 (100.00)	507 (52.92)	
Dead	0 (0.00)	451 (47.08)	
Survival months (mean ± SD)	85.00 ± 40.35	55.55 ± 42.00	<0.001

PTC, papillary thyroid carcinoma; CPTC, classical papillary thyroid carcinoma; FVPTC, follicular variant papillary thyroid carcinoma; ETE, extrathyroidal extension; LNM, lymph node metastasis; CSS, cancer-specific survival; OS, overall survival; SD, standard deviation.

### Propensity Score Matching Compares the Proportion of DM Occurring Among Different Histopathological Subtypes in Children and Adults

1,484 children (≤18 years) and 101,497 adults (>18 years) were included in the study. 41 (2.76%) children had DM, 958 (0.94%) adults had DM (*p*<0.001) (**Table 1**). In patients who had DM, histopathological subtypes were compared between children and adults: CPTC was observed in 36 (87.80%) children and 617 (64.41%) adults, FVPTC was observed in 5 (12.20%) children and 302 (31.52%) adults, other histopathological subtype was only observed in 39 (4.07%) adults (**Table 2**). In CPTC patients, DM was observed in 36 (3.32%) children and 617 (0.93%) adults (*p*<0.001). After the propensity score was matched with ETE and LNM, the proportion of DM was 75% in children, 49.05% in adults(*p*<0.001). In FVPTC patients, DM was observed in 5 (1.29%) children and 302 (0.90%) adults (*p*=0.408). After the propensity score was matched with ETE and LNM, the proportion of DM was 55.56% in children, 49.92% in adults (*p*=1.000) (**Table 3**).

**Table 3:** Comparison of children and adults with distant metastasis in CPTC and FVPTC.

	CPTC				FVPTC			
	No. of patients	With DM [no. (%)]	Without DM [no. (%)]	*P*-value	No. of patients	With DM [no. (%)]	Without DM [no. (%)]	*P*-value
Children	1,083	36 (3.32)	1,047 (96.68)	<0.001	388	5 (1.29)	383 (98.71)	0.408
Adults	66,531	617 (0.93)	65,914 (99.07)		33,474	302 (0.90)	33,172 (99.10)	
after propensity score matching ETE and LNM								
Children	48	36 (75.00)	12 (25.00)	<0.001	9	5 (55.56)	4 (44.44)	1.000
Adults	1,258	617 (49.05)	641 (50.95)		605	302 (49.92)	303 (50.08)	

CPTC, classical papillary thyroid carcinoma; FVPTC, follicular variant papillary thyroid carcinoma; DM, distant metastasis; ETE, extrathyroidal extension; LNM, lymph node metastasis.

### Cut-Off Age for DM in Children With CPTC and FVPTC Calculated by Receiver Operating Characteristic (ROC) Curve and Difference Comparison in the Proportion of Histopathological Subtypes Above and Below the Cut-Off Age

**Figure 1:**
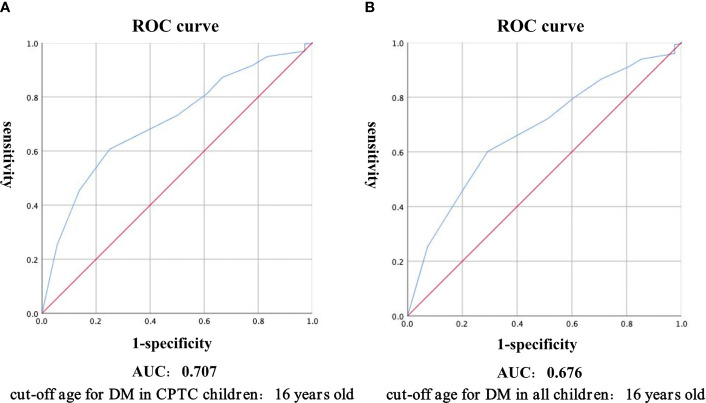
Receiver Operating Characteristic curve was used to calculate the cut-off age for distant metastasis in CPTC children **(A)**, and all children **(B)**. Abbreviations: ROC, receiver operating characteristic; CPTC, classical papillary thyroid carcinoma; AUC, area under curve.

The ROC curve was calculated, which yielded a cut-off age of CPTC children with DM as 16 years old, with the area under the curve of 0.707, sensitivity of 0.750 and specificity of 0.606 (**Figure 1A**). Therefore, we divided patients into young children (2-16 years), adolescents (17-18 years), and adults (>18 years). In CPTC, DM was observed in 31 (5.16%) young children, 5 (1.04%) adolescents and 617 (0.93%) adults (*p*<0.001) (**Table 4-1**). In young children who had DM, 31 (93.94%) patients were CPTC, 2 (6.06%) patients were FVPTC (*p*=0.006). There was no difference between the proportion of CPTC and FVPTC in adolescents and adults who had DM (**Table 4-2**).

**Table 4-1:** Comparison of different age groups with distant metastasis of CPTC.

	No. of patients	With DM [no. (%)]	Without DM [no (%)]	*P*-value	*P*-value		
					(1)	(2)	(3)
2-16years (1)	601	31 (5.16)	570 (94.84)	<0.001	–	<0.001	<0.001
17-18years (2)	482	5 (1.04)	477 (98.96)			–	0.990
Adults (3)	66,531	617 (0.93)	65,914 (99.07)				–

CPTC, classical papillary thyroid carcinoma; DM, distant metastasis.

**Table 4-2:** Comparison of histological subtype in different age groups with distant metastasis of PTC.

	Histological subtype	With DM[no. (%)]	Without DM [no. (%)]	*P*-value
2-16 years	CPTC	31 (93.94)	570 (72.43)	0.006
	FVPTC	2 (6.06)	217 (27.57)	
	No. of patients	33	787	
17-18 years	CPTC	5 (62.50)	477 (74.18)	0.731
	FVPTC	3 (37.5)	166 (25.82)	
	No. of patients	8	643	
Adults	CPTC	617 (67.14)	32,857 (33.16)	0.694
	FVPTC	302 (32.86)	66,229 (66.84)	
	No. of patients	919	99,086	

PTC, papillary thyroid carcinoma; CPTC, classical papillary thyroid carcinoma; FVPTC, follicular variant papillary thyroid carcinoma; DM, distant metastasis.

### Risk Factors of Distant Metastasis in PTC Patients Calculated by Logistic Regression Models

Logistic regression univariate analysis showed that ETE (B 1.824, OR 6.194, 95% CI 2.900–13.228, *p*<0.001), LNM (B 3.330, OR 27.925, 95% CI 3.797–205.354, *p*=0.001), multifocal tumor (B 1.081, OR 2.947, 95% CI 1.410–6.161, *p*=0.004), and the CPTC histopathological subtype (B 1.775, OR: 5.901, 95% CI: 1.400-24.867, *p=*0.016) were risk factors for DM in young children (2-16 years) with PTC. The above meaningful variables for univariate analysis were included in logistic regression multivariate analysis, which showed that ETE (B 1.015, OR 2.759, 95% CI 1.240–6.140, *p*=0.013), LNM (B 2.568, OR 13.039, 95% CI 1.682–101.086, *p*=0.014), and CPTC histopathological subtype (B 1.460, OR 4.308, 95% CI 1.006–18.447, *p*=0.049) were risk factors for DM in young children (2-16 years) with PTC (**Table 5-1**). The CPTC histopathological subtype was not a risk factor for DM in adult patients (>18 years) (**Table 5-2**).

**Table 5-1:** Risk factors of distant metastasis in pediatric patients aged 2-16 years calculated by logistic regression models.

Variables	Univariate analysis				Multivariate analysis			
	B	OR	95% CI	*P*-value	B	OR	95% CI	*P*-value
Gender								
Female	ref							
Male	0.484	1.623	0.739-3.564	0.227				
ETE								
Absence	ref					ref		
Presence	1.824	6.194	2.900-13.228	**<0.001**	1.015	2.759	1.240-6.140	**0.013**
LNM								
Absence	ref					ref		
Presence	3.330	27.925	3.797-205.354	**0.001**	2.568	13.039	1.682-101.086	**0.014**
Foci								
Solitary	ref							
Multifocal	1.081	2.947	1.410-6.161	**0.004**	0.489	1.630	0.752-3.532	0.215
Size								
≤1cm	ref							
>1cm	1.984	7.270	0.986-53.636	0.052				
Ethnicity								
Black	ref							
White	-0.285	0.752	0.172-3.285	0.705				
Other	0.185	1.204	0.224-6.483	0.892				
Histological subtype								
FVPTC	ref					ref		
CPTC	1.775	5.901	1.400-24.867	**0.016**	1.460	4.308	1.006-18.447	**0.049**
Other		0.000		0.999				

B, regression coefficient; OR, odds ratio; CI, confidence interval; ETE, extrathyroidal extension; LNM, lymph node metastasis; FVPTC, follicular variant papillary thyroid carcinoma; CPTC, classical papillary thyroid carcinoma; ref, reference. Bold values indicate statistical significance.

**Table 5-2:** Risk factors of distant metastasis in adult patients calculated by logistic regression models.

Variables	Univariate analysis				Multivariate analysis			
	B	OR	95% CI	*P*-value	B	OR	95% CI	*P*-value
Gender								
Female	ref					ref		
Male	1.133	3.104	2.732-3.527	**<0.001**	0.823	2.277	1.999-2.595	**<0.001**
ETE								
Absence	ref					ref		
Presence	2.147	8.563	7.498-9.780	**<0.001**	1.501	4.486	3.866-5.206	**<0.001**
LNM								
Absence	ref					ref		
Presence	1.850	6.362	5.563-7.275	**<0.001**	1.119	3.062	2.629-3.566	**<0.001**
Foci								
Solitary	ref							
Multifocal	0.400	1.491	1.313-1.694	**<0.001**	-0.091	0.913	0.800-1.041	0.174
Size								
≤1cm	ref					ref		
>1cm	1.619	5.048	4.147-6.146	**<0.001**	0.849	2.337	1.904-2.868	**<0.001**
Ethnicity								
Black	ref							
White	-0.165	0.848	0.659-1.090	0.198				
Other	0.274	1.315	0.986-1.756	0.063				
Histological subtype								
FVPTC	ref					ref		
CPTC	0.028	1.028	0.895-1.181	0.694	-0.477	0.621	0.537-0.718	**<0.001**
Other	1.081	2.948	2.104-4.132	**<0.001**	-0.103	0.902	0.637-1.278	0.562

B, regression coefficient; OR, odds ratio; CI, confidence interval; ETE, extrathyroidal extension; LNM, lymph node metastasis; FVPTC, follicular variant papillary thyroid carcinoma; CPTC, classical papillary thyroid carcinoma; ref, reference. Bold values indicate statistical significance.

## Discussion

Thyroid cancer is the most rapidly increasing cancer in the United States (16). The incidence rates of pediatric thyroid cancer (patients younger than 20 years) increased more rapidly from 2006 to 2013 than from 1973 to 2006 (2). The incidence of DM is significantly more frequent in children (<19 years) than in adults, but the prognosis is generally good (3) with unclear reasons. By analyzing the demographic information and clinicopathological characteristics of a large number of PTC patients, this study confirmed that the incidence of DM in pediatric PTCs (≤18 years) was higher than that in adults, and the incidence of pediatric CPTCs (≤18 years) was also higher than that in adults. We compared the proportion of children (≤18 years) and adults with DM in CPTC and FVPTC respectively by propensity score matching. The results showed that in CPTCs, children had a higher rate of DM than adults. However, in FVPTCs, there was no difference in the incidence of DM between children and adults. We also used ROC curve to calculate the cut-off age for DM in CPTC children, and compared the difference in the proportion of histopathological subtypes above and below the cut-off age. According to our calculations, CPTCs had a higher rate of DM than FVPTCs in both children (≤18 years) and young children (2-16 years). There was no difference in DM rate between CPTC and FVPTC in adolescents (17-18 years) and adults. These findings further validated the correlation between CPTC and DM in young children. Finally, logistic regression models revealed that ETE, LNM, and CPTC histopathological subtype were risk factors for DM in young children aged 2-16 years. However, CPTC histopathological subtype was not a risk factor for DM in adolescents aged 17-18 years and in adults aged older than 18 years.

PTC is more common in female than in male in a research including patients aged 0-24 years and the incidence of thyroid cancer increased with age (17). For pediatric population, difference in gender starts just above age 10, with increasing distinction above age 15 (18). There had been previous studies dividing thyroid cancer patients into pediatrics (<13 years) and adolescents to compare the metastasis and disease progression (19). Within the pediatric group under 18 years of age, special attention should be paid to male patients under 15 years old, as they are associated with a more advanced disease at diagnosis (5). Tumors were generally infiltrative in patients younger than 15 years (20). Therefore, we believe that it is necessary to take the age into consideration to divide young children separately from adolescents during the development and disease progression of PTC. We calculated the cut-off age for DM in children as 16 years old by ROC curve, with the area under the curve of 0.676, sensitivity of 0.602 and specificity of 0.707 (**Figure 1B**), and we found that adolescent patients aged 17-18 years performed similar to adult patients regardless of the proportion of DM or the distribution of histopathological subtypes, while young children aged 2-16 years exhibited significantly different features from adolescent patients aged 17-18 years and adult patients. Therefore, further studies are needed to provide better individualized treatment for pediatric PTC patients.

A previous study had shown that CPTC and FVPTC were different in driving somatic genetic alterations and cell signal transduction, which leaded to the poor differentiation and strong invasiveness of CPTC (21). CPTC was a strong predictor of high recurrence risk and high cancer-specific mortality, with a worse prognosis than FVPTC (9). In a study including 163 patients aged less than 18 years diagnosed as PTC, BRAF mutations and RET and NTRK fusions were detected mainly in CPTCs (20). The presence of a BRAFV600E mutation was reported to be correlated significantly with the need for a second treatment during the follow-up in patients under 18 years of age, and BRAF mutations might be associated with more aggressive clinical features and a higher risk of recurrence or persistence of disease in the pediatric population (5). Furthermore, the fusion-driven tumors, in general, displayed a lower thyroid differentiation score than mutation-driven samples (≤18 years), suggesting that gene fusion-positive pediatric PTCs are less differentiated (22). Besides, fusion gene-positive pediatric PTC cases (6-20 years) had more aggressive disease with more frequent extrathyroidal extension and lymph node and distant metastases than patients without fusion genes (23). This finding prompted us to speculate that the poor outcome in classical PTCs compared to FVPTCs was largely attributable to higher proportion of BRAF V600E mutations and RET and NTRK fusions in the former group. Clinically, the prevalence of high-risk parameters was significantly different among the two subtypes. Risk factors including ETE, LNM, stages III/IV, disease recurrence, radioiodine treatment, as well as mortality were lower in FVPTC (24). After studying a subgroup of FVPTC with an intact tumor envelope and very good prognosis, it is referred to as “non-invasive follicular thyroid tumor with papillary features” (NIFTP) and is classified as a non-cancerous tumor (25). In conclusion, the biological behaviors and disease prognosis of CPTC and FVPTC differ significantly, and they should be distinguished in diagnosis and treatment.

It has been reported that CPTCs were less represented in patients aged less than 15 years than in patients aged 15-18 years, while FVPTCs occurred more frequently in the former group (20). However, our results showed that the proportion of CPTCs was higher than that of FVPTCs, both in children aged ≤18 years and in young children aged 2-16 years regardless of with or without DM. The reason for this difference may be that our study is based on a large sample size, but the previous study included only 163 samples. In the other hand, our logistic regression analysis showed that tumor diameter > 1 cm was not a risk factor for DM in patients aged 2-16 years (*p*>0.05). This indicates that although children, when compared to adults, had larger primary tumors (26) and the tumor diameter is related to the poor prognosis of FVPTC (9), but tumor diameter is not a risk factor for DM in patients aged 2-16 years, which may be one of the reasons why the proportion of FVPTCs in patients aged 2-16 years with DM is lower than that of CPTCs.

The molecular biological characteristics of the pathogenesis of thyroid cancer in children and adults may explain the differences in clinical manifestations and prognosis (27). Despite this, the clinical assessment and treatments used in pediatric thyroid cancer are the same as those implemented for adults (18). Histopathological subtypes have recently been shown to play an important role in determining the persistence and/or recurrence of disease (28). Our data show that the incidence of DM in young children (2-16 years) with CPTC is significantly higher than that in adolescents (17-18 years) and adults, and that the same histopathological subtype presents different clinical and histopathological features in different age groups. ETE, LNM, and CPTC subtype are risk factors for DM in pediatric patients aged 2-16 years. We suggest that the histopathological subtypes of CPTC and FVPTC should be classified and managed separately in patients aged 2-16 years to cope with the persistent or recurrent risk of disease, but further studies are needed to expand our findings, which may guide therapeutic strategies.

This study has certain limitations. First, it was a retrospective analysis based on the SEER database. There was an inherent selection bias. To control for selection bias, we adopted a rigorous scientific study design, clarified the inclusion criteria and exclusion criteria of subjects, and unified the disease diagnosis. Second, not all data are available from the SEER database, such as patient recurrence information, not allowing analysis of the subsequent DM occurrence in different histopathological subtypes. Third, the use of stratified analysis in this study would cause statistical deviation due to the small number (41 cases) of pediatric patients with DM. Finally, data in this study were extracted from the SEER database from 2004 to 2015, but the 2017 WHO classification introduced the NIFPT terminology for encapsulated FVPTCs. As a result, a minority of the “FVPTCs” reported in the SEER database may in fact be NIFPTs. It might affect the differences in outcome between bona fide FVPTCs vs CPTCs within an acceptable range.

However, our study is the first with a large sample to investigate the role of common histopathological subtypes in determining DM of pediatric PTC and the reasons of the high DM incidence in children. We propose that pediatric PTC patients should be divided into patients aged 2-16 years and patients aged 17-18 years. CPTC patients aged 2-16 years might be treated more aggressively. Further studies in the future may be helpful to guide the treatment strategies for PTC in pediatric patients.

In conclusion, this study showed that in CPTC, the incidence of distant metastasis in young children (2-16 years) was significantly higher than that in adolescents (17-18 years) and adults (>18 years). There was no such difference among patients with FVPTC. In patients with distant metastasis aged 2-16 years, CPTC patients had a higher rate of DM than FVPTC patients. Extrathyroidal extension, lymph node metastasis, and CPTC histopathological subtype were risk factors for distant metastasis in young children aged 2-16 years.

## Data Availability Statement

The raw data supporting the conclusions of this article will be made available by the authors, without undue reservation.

## Author Contributions

XZ, ZW, ZG, JX, MC, WS, LH, WD, JH, DZ, CL, TZ, LS, PZ, and HZ contributed to this study. ZW contributed to the conception and design of this study. XZ collected data. XZ and ZG performed the statistical analysis. XZ and ZW drafted and wrote the manuscript. All authors contributed to the article and approved the submitted version.

## Funding

This research was supported by the Natural Science Foundation of Liaoning Province (grant number 2020-MS-143), the Natural Science Foundation of Liaoning Province (grant number 20180530090), the National Natural Science Foundation of China (grant number 81902726), and the China Postdoctoral Science Foundation (grant number 2018M641739).

## Conflict of Interest

The authors declare that the research was conducted in the absence of any commercial or financial relationships that could be construed as a potential conflict of interest.

## Publisher’s Note

All claims expressed in this article are solely those of the authors and do not necessarily represent those of their affiliated organizations, or those of the publisher, the editors and the reviewers. Any product that may be evaluated in this article, or claim that may be made by its manufacturer, is not guaranteed or endorsed by the publisher.

## Abbreviations

AUC, area under curve; B, regression coefficient; CI, confidence interval; CPTC, classical papillary thyroid carcinoma; CSS, cancer-specific survival; DM, distant metastasis; ETE, extrathyroidal extension; FVPTC, follicular variant papillary thyroid carcinoma; LNM, lymph node metastasis; OR, odds ratio; OS, overall survival; PSM, propensity score matching; PTC, papillary thyroid carcinoma; RAI, radioactive iodine; ROC, receiver operating characteristic; SD, standard deviation.
